# Effects of the Probiotic *Enterococcus faecium* on Muscle Characteristics of Chickens

**DOI:** 10.3390/life12111695

**Published:** 2022-10-25

**Authors:** Elke Albrecht, Rudolf Zitnan, Viera Karaffova, Viera Revajova, Michaela Čechová, Martin Levkut Jr., Monika Röntgen

**Affiliations:** 1 Research Institute for Farm Animal Biology (FBN), Institute of Muscle Biology and Growth, 18196 Dummerstorf, Germany; 2 National Agriculture and Food Centre, Research Institute for Animal Production Nitra, 95141 Lužianky, Slovakia; 3 Department of Morphological Disciplines, University of Veterinary Medicine and Pharmacy, 04181 Košice, Slovakia

**Keywords:** poultry, probiotics, *Enterococcus faecium*, muscle tissue, *IGF-1*, myogenic regulatory factors, PAX7, antibiotics replacement

## Abstract

The use of antibiotics in farm animals is one of the main reasons for the development of resistant bacterial strains (e.g., zoonotic pathogens). Therefore, save alternatives are needed. Here, we examined how post-hatch application (day one to seven of life) of the probiotic *Enterococcus faecium* AL41 (EF) affects the development and tissue properties of the broiler pectoralis major muscle (PM). Expression of regulators, namely *IGF-1*, *PAX7*, and *MYF5*, was also investigated. At day 1 (*n* = 6), and days 5, 8, and 12 (*n* = 10), muscle samples were taken from control and EF supplemented chicks. From day 5 on, myonuclei number per fiber was elevated in EF chicks. Improved capillarization (from day 8), larger myofibers, increased body and PM weights (day 12) were found in the EF group. Part of our findings is explainable by higher intramuscular expression of *IGF-1* and lower *MYF5* expression in EF birds. In both groups *IGF-1* expression decreases with age, thereby increasing the cellular myogenic potential. However, a strong increase in *PAX7* expression and more PAX7-positive nuclei were found in EF chicks at day 12. We conclude that EF supplementation improves PM growth and health due to positive effects on bioavailability and fusion capacity of SATC progeny and better tissue perfusion.

## 1. Introduction

Over the last few decades, the consumption of chicken meat has increased at the same time as consumer demands regarding its quality and safety have been increasing. [1,2].

To meet the growing demand for chicken meat, traditional forms of husbandry have over time been replaced by scaled industrial production systems in which the animals live mostly or completely indoors during their life span. Associated negative effects relate in particular to the intestinal microbiome [3]. Chicks hatched in incubators show a delayed colonization of the gut with commensal bacteria [4,5] and, later on, density and complexity of the population is reduced [6]. Consequently, a lower resistance to pathogens, disturbed nutrient absorption, higher mortality rates, and production costs (mainly due to a reduced feed conversion rate) have been increasingly observed [7,8].

For a long time, attempts were made to solve these problems by regular application of low, sub-therapeutic amounts of antibiotics to animals [9]. These antimicrobial growth promoters (AGPs) reduced the occurrence of gastrointestinal infections and promoted feed efficiency, growth performance, and, thereby, meat mass [7,10,11].

However, the intensive use of antibiotics at sub-therapeutic doses has contributed to development of resistant bacterial strains in commensals and zoonotic pathogens [12,13]. Thus, to prevent negative effects on human health, AGP’s have been banned in the European Union since 2006. However, the use of therapeutic antibiotics for farm animals increased significantly thereafter [3,14].

Consequently, there is growing global interest in effective and save AGP replacements enabling human and animal health, food security, and efficiency of production. Various alternatives have been proposed and tested, among them the application of protective microbiota [15,16] such as probiotics (live microbial feed supplements).

Per definition, probiotics are “products that contain sufficient values of viable bacteria that can improve the intestinal microbial balance of the host resulting in detectable beneficial health effects” [16,17]. Various bacteria such as *Lactobacillus* spp., *Bacillus* spp., *Bifidobacterium* spp., *Streptococcus* spp., and *Enterococcus* spp. are known to have probiotic properties when added to poultry diets [5,7,8,10,18,19,20]. In addition, beneficial effects on poultry growth performance and on feed utilization efficiency have been reported for various probiotics [5,7,8,20,21] but results show a higher variability than with AGPs [10,22].

The probiotic species *Enterococcus faecium* (EF) suppress colonization and proliferation of enteric bacteria among them *Salmonella* Enteritidis (SE) [15,19,23,24,25] and *Listeria innocua* HPB13 [16]. In addition, EF strains are able to produce antimicrobial substances, so-called bacteriocins [16,19,25]. However, in contrast to antibiotics, the killing spectrum of EF bacteriocins is relative narrow and their toxicity is only directed against close relatives of the producing strain [25,26]. Early post-hatch administration of EF to chicken reduced cecal colonization with pathogenic SE, stimulate innate and acquired immune responses [16,27,28], stabilized the protective function of the intestinal epithelial barrier, and promotes small intestine development [20,24,25,29]. Thus, at least in part, a higher absorptive capacity for nutrients, specifically for glucose, will contribute to the positive effects of EF strains on growth performance [20,29].

However, economically, the growth and quality of the skeletal muscle is most significant for broiler meat production [30]. Modern broiler strains are selected for fast muscle growth and, in particular, an accelerated growth of the pectoralis muscle (PM) [31].

The number of muscle fibers is already established at hatch [32]. Therefore, muscle hypertrophy occurring by the accretion of protein and new myonuclei to existing myofibers is responsible for post-hatch muscle growth [33]. New myonuclei for this process originate from so-called satellite cells (SATC) [34,35,36]. The majority of SATC is quiescent in adult animals, and can be found adjacent to myofibers in a niche underneath their basement membrane [33]. In contrast, SATC show a high proliferative capacity in growing broiler muscles reaching a peak activity already between days two and four after hatching [37,38]. After differentiation, most SATC fuse to existing myofibers to enlarge their diameter [30,34,39] whereas some SATC self-renew to replenish their own population [40,41]. A group of myogenic genes regulates SATC bioavailability and their molecular and functional properties. During quiescence, paired box protein 7 (*PAX7*) and the determination gene myogenic factor 5 (*MYF5*) are expressed [41,42,43]. In activated, proliferating cells (myoblasts) determination protein 1 (*MYOD*) is induced [44,45]. Downregulation of *PAX7* and induction of myogenin (*MYOG*) characterize the process of early differentiation (myocytes) whereas sarcomeric myosin heavy chain (*MYHC*) is expressed in terminally differentiated cells [44,45].

However, the molecular and functional phenotype of SATC can also be affected by environmental stimuli (e.g., nutrients or growth factors) [46,47]. Short-term reductions in SATC mitotic activity during the early post-hatch period (e.g., due to feed deprivation or high ambient temperature) decrease muscle size at maturity, showing its critical role for maintaining SATC activity and subsequent muscle growth [33,38,48]. In our previous study [49], early post-hatch SE infection induced dramatic negative effects in muscle tissue, among them permanently reduced myofiber cross sectional area (FCSA) and disturbed capillarization. However, preventive application of the EF strain AL41 completely restored blood supply pointing to more direct effects of EF AL41 on muscle development [49]. To our knowledge, there is no experimental work on the effect of oral probiotics on processes of early postnatal muscle development. However, we think that such information is prerequisite for commercial application of probiotics.

Therefore, in addition to morphological and biochemical properties of muscle tissue, the current study examines if and how selected regulatory systems of muscle development will be affected by a seven-day post-hatch EF AL41 treatment. Our results show clear positive effects of probiotic application on daily weight gain, body and PM weight accompanied by improved hypertrophic myofiber growth and capillarization. In addition, our study provides new findings showing that the intramuscular IGF-1 system and main regulators of SATC functionality, namely the myogenic regulatory factors *PAX7* and *MYF5*, are modulated by EF treatment giving a possible explanation for positive effects of the probiotic on growth performance.

## 2. Materials and Methods

### 2.1. Animals

The chicks were handled and killed according to the Slovak state regulations. The experiment was approved by the Ethics Committee of the University of Veterinary Medicine and Pharmacy, Košice, Slovakia, and by the Committee for Animal Welfare of the Ministry of Agriculture of the Slovak Republic (permission number 1184-3/2020-220).

Eighty one-day-old COBB 500 male chicks were randomly divided into two groups, a control group (Con) and a treatment group, respectively. Birds of the treatment group were inoculated with the probiotic strain *Enterococcus faecium* (*E. faecium*) AL41 (CCM 8558) as described by Zitnan et al. [49]. The probiotic strain *E. faecium* AL41 (EF) was grown as described by Letnická et al. [50]. From the first to the seventh day of the experiment, a suspension with 10^9^ colony-forming units of EF in 0.2 mL PBS was applied to every chicken of the treatment group *per os*. To simulate the same stress to chicken of the control group, an equal volume of PBS only was applied with a Pasteur pipette. The chicks were placed in hardwood pens with wood shavings and fed a standard BR-1 compound feed. The diet composition has been published in [51]. Access to water was provided *ad libitum*. Housing conditions were chosen according to Cobb-Vantress Inc. guidelines [52]. Prior to the start of the experiment, faecal control samples were taken from the chickens for microbiological examination.

For the present experiments, six birds per group were slaughtered at the beginning of the experiment (day one of life). Subsequently, 10 birds per group were slaughtered at days 5, 8, and 12 of life. Body weights were recorded at slaughter and pectoralis muscle (PM), liver, stomach, and intestine were weighed and collected. Muscle tissue was cut into pieces that were separately frozen in liquid nitrogen for biochemical and histological analyses. Samples from four randomly selected animals per group were immediately placed in RNA Later solution (Qiagen, Manchester, UK) for gene expression analyses and stored at −70 °C until further processing.

### 2.2. Biochemical Analyses

Frozen muscle samples were prepared as described by Zitnan et al. [49] for determination of DNA, RNA, and protein content, as well as enzyme activities. All muscle homogenates were diluted (1:20), then centrifuged at 14,000× *g* (15 min, 4 °C), and the resulting supernatants were kept on ice. All enzyme activity assays and the determination of the protein content were conducted the same day. The content of DNA and RNA in diluted supernatants (1:2 and 1:50, respectively) were measured using Hoechst 33258 and SYBR Green II, against respective standards (all Sigma-Aldrich, Steinheim, Germany) in an Flx-800-I microplate fluorescence reader (Bio-Tek Instruments, Bad Friedrichshall, Germany) as described earlier [49]. The protein content was analysed in 1:50 diluted supernatants according to Peterson [53]. Enzyme activities of creatine kinase (CK, EC 2.7.3.2), isocitrate dehydrogenase (ICDH, EC 1.1.1.42), and lactate dehydrogenase (LDH, EC 1.1.1.28) were determined as described by Lösel et al. [54]. Supernatants from 1-day-old chicken were diluted 1:100 and all others 1:200 for determination of CK activity using a commercial kit (CK-NAC-Hit kit, IFCC method, BIOMED Labordiagnostik GmbH, Oberschleißheim, Germany) adapted for microplates. Activities of ICDH and LDH were analysed as described by Zitnan et al. [49] in undiluted or diluted (1:5 at d 1, 1:15 at d 5, 1:30 at d 8 and 12) supernatants, respectively. A Spectramax Plus384 spectrophotometer/plate reader (Molecular Devices, Sunnyvale, CA, USA) was used for quantification.

### 2.3. Histomorphological Analysis

PM samples were cut 10 µm thick at −20 °C using a cryostat microtome Leica CM3050 S (Leica, Wetzlar, Germany). Sections were stained and analysed as described by Zitnan et al. [49] using standard protocols for hematoxylin/eosin (HE) [55] and for capillaries with eosin and alkaline phosphatase (EAP) [56]. At least three randomly selected images per sample were taken using an Olympus BX43 microscope (Olympus, Hamburg, Germany) equipped with a UC30 colour camera and Cell^D imaging software (OSIS, Münster, Germany). Muscle fiber und nuclei number were counted in HE stained slides using the interactive measurement function of Cell^D. The muscle fiber size, capillary size and density (number per mm^2^) were determined in EAP-stained slides with a self-made macro program as described by [49]. The ratio between counted numbers of myonuclei and muscle fibers was calculated as nuclei/fiber. The size of muscle fibers was an estimated muscle fiber cross sectional area (FCSA) determined by dividing the analyzed area by the number of muscle fibers. The total area analysed per sample was about 25,900 µm^2^ at d 1, 36,970 µm^2^ at d 5, 60,263 µm² at d 8, and 94,567 µm^2^ at d 12.

### 2.4. Gene Expression Analysis

Gene expression of *IGF1*, *PAX7, MYF5*, and *GAPDH* (glyceraldehyde-3-phosphate dehydrogenase) was determined. Based on confirmed expression stability using the Bestkeeper program, *GAPDH* was selected as the reference gene. About 20 mg muscle tissue was used for RNA purification and transcription as described by Karaffová et al. [57].

Amplification and detection of target products were performed using the CFX 96 RT system (Bio-Rad, Hercules, CA, USA) and Maxima SYBR Green qPCR Master Mix (Thermo Scientific, Waltham, MA, USA). Subsequent RT-qPCR to detect relative mRNA abundance was performed for 36 cycles under the following conditions: initial denaturation at 95 °C for 2 min, subsequent denaturation at 95 °C for 15 s, annealing (as listed in Table 1) and extension step 2 min at 72 °C. A melting curve from 50 °C to 95 °C with readings at every 0.5 °C was recorded for each individual RT-qPCR plate. Analysis was performed after every run to ensure a single amplified product for each reaction. All reactions for qPCR were carried out in duplicate. The primer sequences, annealing temperatures and times for each primer used for RT-qPCR are listed in Table 1. All primer sets allowed cDNA amplification efficiencies between 94% and 100%. It could be confirmed that the efficiency of amplification for each target gene (including GAPDH) was essentially 100% in the exponential phase of the reaction, where the quantification cycle (Cq) was calculated. The Cq values of the studied genes were normalised to an average Cq value of the reference gene (ΔCq), and the relative expression of each gene was calculated as 2^−ΔCq^. The relative gene expression in muscle of control chicken at day one was set to 1 to calculate fold changes for the values at the other ages and in the EF group.

### 2.5. Immunohistochemistry and Image Analysis

Muscle tissue of each two animals per age and treatment group were randomly selected in addition to all samples of 12-day old chickens for immunohistochemistry and quantification of PAX7 positive cells. Tissue was cut 10 µm thick with a cryostat microtome Leica CM3050 S (Leica, Wetzlar, Germany). Fixation with 4% paraformaldehyde in PBS, was followed by washing three times for 10 min with PBS including Tween 20 (PBST) and a blocking step with 10% normal goat serum (NGS) and 10% BSA in PBS for 30 min. Incubation over night with the primary antibody against PAX7 (Developmental Studies Hybridoma Bank, Iowa, USA; 1:50 in PBS with 1% BSA) was followed by three times wash with PBST and incubation with the secondary antibody Alexa Fluor 594 goat anti-mouse IgG H & L (1:500, Thermo Fischer Scientific, Waltham, MA, USA) for 1.5 h at room temperature. Slides were washed three times with PBS for 5 min and covered with Roti Fluor Care DAPI (Roth, Karlsruhe, Germany) to counterstain all nuclei. A negative control, omitting the primary antibody, was processed to detect unspecific binding of the secondary antibody. No unspecific staining was observed. Immunofluorescence of PAX7 and DAPI-stained nuclei was detected using a Nikon Microphot SA fluorescence microscope (Nikon, Duesseldorf, Germany) equipped with a CC-12 color camera (OSIS, Münster, Germany) and Cell^F image analysis software (OSIS). A macro program was developed to count the total number of nuclei (stained blue) and the number of PAX7-positive nuclei (stained red) as follows. The blue image of total nuclei was opened, a shading correction was performed, the blue channel extracted, followed by a mean filter. A region of interest (ROI) was defined. An ultimate erosion followed by two dilatation steps enabled separation of closely located nuclei. Nuclei were then detected by a threshold operation and counted, after manual correction/deletion of false detected particles, such as artifacts. Afterwards, the red image of PAX7-positive nuclei was opened, the contrast was enhanced, the red channel extracted and the nuclei detected by threshold operation. False detected particles were deleted interactively and the results were saved. Ten images per animal were analyzed with a total area of about 2.37 mm^2^. For each animal, the number of nuclei per mm², the number of PAX7 nuclei per mm^2^, the percentage of PAX7 nuclei, the number of nuclei per muscle fiber and the number of PAX7 nuclei per muscle fiber were calculated.

### 2.6. Statistical Analysis

Statistical analysis was performed using the ANOVA model of SAS statistical software (Version 9.4, SAS Inst., Cary, NC, USA). Data was analyzed with the MIXED procedure with fixed factors treatment (Con and EF), age (1, 5, 8, and 12 d), and their interaction. All data are presented as least square means (LSM) and standard error (SE). As a post-hoc test, the Tukey-Kramer correction was used. The SLICE statement of the MIXED procedure was applied to enable the partitioned analysis of the effect of the treatment within the same age. A *p*-value of <0.05 was considered statistically significant and 0.05 < *p* < 0.1 a trend.

## 3. Results

### 3.1. Weight Development

In both experimental groups, slaughter weight as well as weights of the PM and inner organs increased from day 1 to day 12 of life (*p* < 0.001, Table 2). The fast growth during the first days of life was reflected by an increasing daily gain from day five to day eight of age (*p* < 0.001, Table 2). The growth slowed down thereafter, as indicated by a lower daily gain for the 12 day group. Over all ages, the daily gain was significantly higher in EF compared to Con chicks (19.8 vs. 18.0 g/d, *p* > 0.001). Control and EF-supplemented chicks developed similarely up to the eighth day of life. However, at day 12 of age, EF chicks had a higher slaughter (*p* = 0.015) and PM (*p* = 0.009) weight than Con chicks (Table 2). The livers of Con chicks were heavier at the 5th and 12th days of age (*p* < 0.001 and *p* = 0.038, respectively) whereas the stomach weight was greater in EF chicks at day 5 of age (*p* = 0.009). Regarding the intestinal weights no group differences (*p* > 0.05) were found.

### 3.2. DNA, RNA, Protein and Muscle Enzymes

Table 3 displays changes of DNA, RNA, and protein amounts in PM between day 1 and 12 of age in control and EF-treated chicks. In both groups, total DNA, RNA, and protein increased (*p* > 0.05). The DNA/protein and RNA/protein ratios were highest at d 5 in both groups (*p* < 0.05).

Activity of CK and LDH increased with age (*p* < 0.05), whereas the ICDH activity decreased (*p* < 0.05) in both groups. No group differences in the activity of investigated muscle enzymes were observed. The increase of LDH/ICDH ratio with age was also similar in both groups.

### 3.3. Muscle Structure

**Figure 1:**
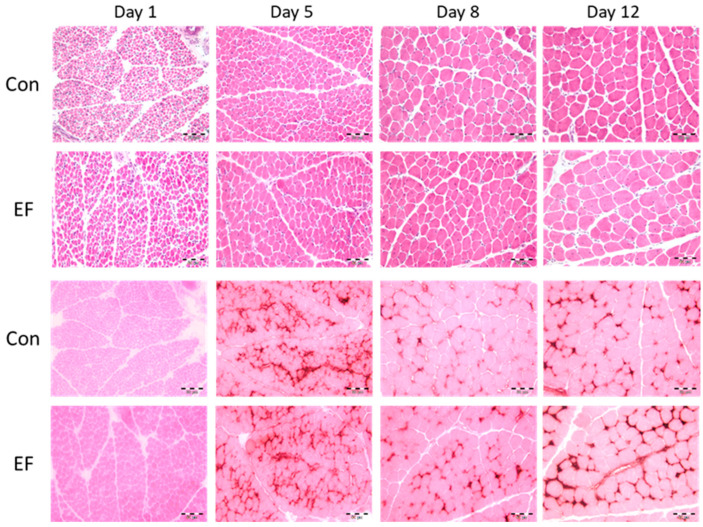
Muscle structure of control (Con) and *E. faecium* AL41 (EF) supplemented chicks. Representative *pectoralis* muscle histological sections of chicks at 1, 5, 8, and 12 d of age, stained with hematoxylin/eosin (HE, upper panel) or eosin/alkaline phosphatase (EAP, lower panel) as used for image analysis. Scale bars represent 50 µm.

Next, myonuclei density, fiber cross sectional area (FCSA), myonuclei per fiber and capillarization of PM were determined in control and EF treated chicks. The results are summarized in Figure 1 and Table 4.

In both groups, the FCSA increased with age (*p* < 0.001). However, at day 12 of age larger muscle fibers were found in EF chicks (*p* < 0.001). As expected, the number of nuclei per mm^2^ decreased (*p* < 0.001) from day 1 to day 12 post-hatch in Con and EF chicks. At day 1, myonuclei density was higher in the Con group whereas it was lower in Con than EF chicks at day 5. In addition, the number of myonuclei per muscle fiber was higher in EF group chicks at days 5, 8, and 12 (*p* < 0.05).

Due to the immaturity of the muscle tissue on day one of life, the EAP staining did not work. Therefore, it was impossible to investigate the capillarization at this age. Control chicks had more capillaries per mm^2^ and a higher number of capillaries per myofiber at day five of age. At 12 d of age, capillaries in the PM of EF supplemented chicks had a larger CCSA and thus, the area covered by capillaries was larger than in the control group (*p* < 0.05).

### 3.4. Gene Expression

**Figure 2:**
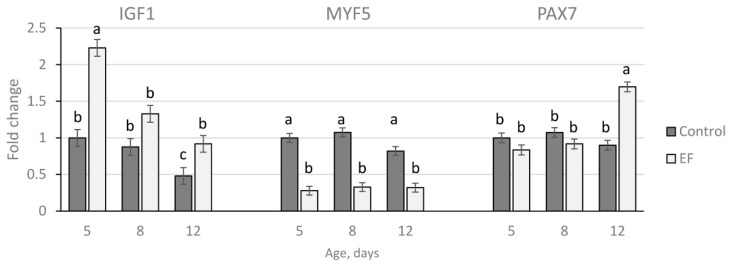
Gene expression of *IGF1*, *MYF5*, and *PAX7* in *pectoralis* muscles of untreated control and *E. faecium* AL41 (EF) supplemented chicks. Results at each time point are fold changes of expression of control at five days of age. ^a–c^ different letters indicate significant differences among groups and time points at *p* < 0.05.

Gene expression, calculated as fold changes to Con at day five of age, is presented in Figure 2. A reduction of *IGF1* mRNA was observed from the 5th to the 12th day of life in both groups (*p* < 0.05). The relative expression for *IGF1* was higher in the EF group compared to the control at day 5 and day 12 (*p* < 0.001). In contrast, *MYF5* expression was relatively constant over age in both groups (*p* > 0.1). The mRNA abundance was markedly down-regulated in the EF group (*p* < 0.001) at days 5, 8, and 12 of age. The transcription factor *PAX7* was up-regulated in the EF group (*p* < 0.001) at day 12.

### 3.5. PAX7 Protein Abundance in Pectoralis Muscle

**Figure 3:**
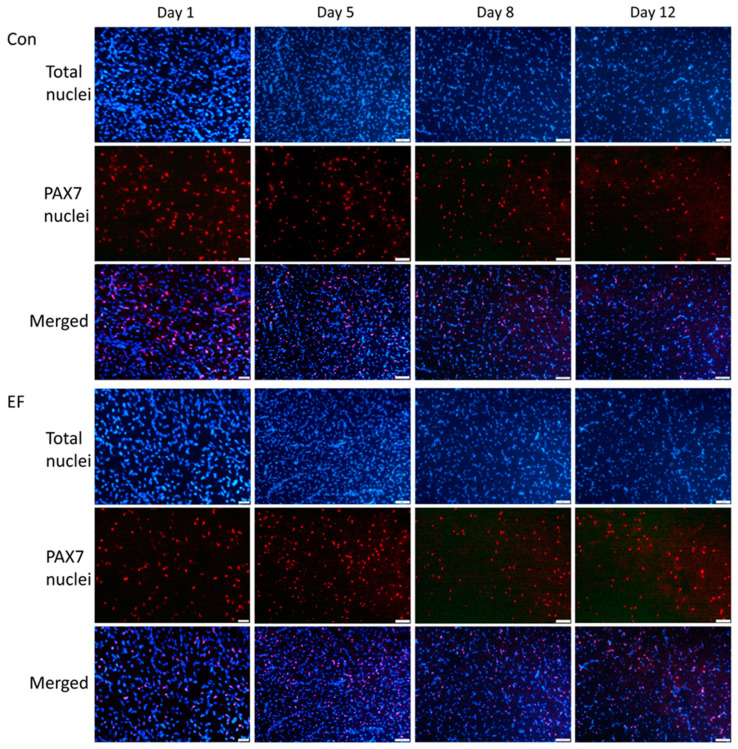
Immunefluorescence of PAX7 in *pectoralis* muscle cross sections of untreated control (Con) and *E. faecium* AL41 (EF) supplemented chicks. Total nuclei were counterstained with DAPI (blue). Merged images indicate nuclear localization of PAX7. Scale bars represent 20 µm at day 1 and 50 µm at other ages.

**Figure 4:**
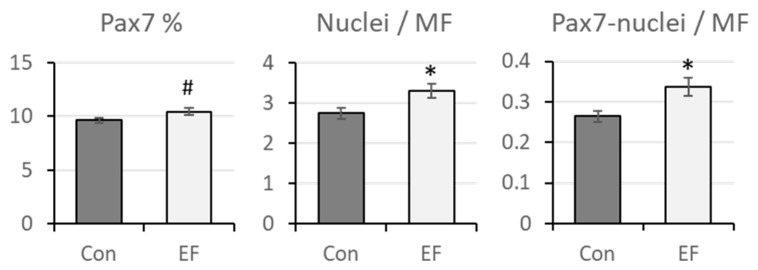
Quantification of total and PAX-7 positive nuclei in *pectoralis* muscle of untreated control (Con) and *E. faecium* AL41 (EF) supplemented chicks at 12 d of age. The percentage of Pax7-positive nuclei, the total number of nuclei per myofiber and the number of Pax7-positive nuclei per myofiber are given. * indicate significant differences between the groups at *p* < 0.05 and # indicates a trend at *p* = 0.09.

Increased gene expression of *PAX7* in EF chicks at 12 d of age was further elucidated on protein level. Immunohistochemistry was applied to detect PAX7-positive nuclei in muscle sections as shown in Figure 3. As expected, PAX7 staining was observed in nuclei, verified by co-localization with DAPI nuclear staining, and PAX7-positive nuclei were observed close to muscle fibers. The proportion of PAX7-positive nuclei and the number of Pax7-positive nuclei per myofiber were quantified in muscle sections of chicks at 12 d of age solely (Figure 4), since the difference in gene expression between Con and EF chicks was observed only in this age (Figure 2). There was a trend for a greater percentage of PAX7-positive nuclei in EF chicks (*p* = 0.09). The numbers of nuclei per muscle fiber and PAX7-positive nuclei per muscle fiber were both higher in EF chicks (*p* = 0.021 and 0.013, respectively).

## 4. Discussion

Whereas probiotics regularly show positive health effects, its growth promoting effects are often inconsistent [22]. As PM growth is a main component of performance in broiler chickens, we aimed at getting information on the possible direct effects of the poultry-specific probiotic *E. feacium* AL41 concerning its development and tissue properties. SATC and their progeny are essential for post-hatch muscle development. Therefore, in addition to biochemical properties, we also examined myogenic factors (*Pax7* and *MYF5*) and *IGFI* known as important regulators of SATC bioavailability and functionality [41,44,47].

As expected, body and PM weight increased strongly over the experimental period and the contribution of PM to BW increased from about 4 to 24% within twelve days. Accelerated growth of the breast muscle is a characteristic of poultry strains selected for meat production [31]. In our study, this is reflected by a fast increase of total amounts of DNA, RNA, protein and CK in muscle samples from both groups. In addition, the overall RNA/DNA ratio amounts to about 5.9 reflecting the high overall transcriptional activity of cells in broiler PM or in other words, its high capacity to accumulate ribosomes [61]. For example, The RNA/DNA ratio of liver cells, another cell type showing high rates of protein synthesis, amounts to 4.7, whereas those of thymocytes amounts to 0.3 [61]. However, daily weight gain (19.8 g/d) and relative increases in PM weight (day 1 to 8: 21.7; day 8 to 12: 0.68) were higher in EF compared with Con (18.0 g/d, day 1 to 8: 19.5; day 8 to 12: 0.64) chicks. Thus, at the end of the study period, body weight and PM weight are higher in EF-treated animals. Exclusively in muscle samples from EF-treated chicks, we found a higher amount of total RNA at day 12 of age, again indicating a higher anabolic potential after probiotic supplementation. As 85% of total RNA is ribosomal, higher levels represent an elevation in the machinery necessary for mRNA translation into protein [62].

On the one hand, the age-dependent increase in total CK activity of PM indicates a higher proportion of myofiber proteins [63]. However, muscular CK is predominantly expressed in fetal and adult fast fibers [64,65]. Thus, the observed increase of CK could reflect processes of muscle maturation including the conversion of slow to fast myofibers, the predominant fiber type in the adult PM [66]. In accord, ICDH activity, a marker of oxidative muscle metabolism decreased whereas LDH activity, a measure of glycolytic muscle metabolism, increased with age. The resulting increase of the LDH/ICDH ratio, confirms an age-dependent shift to the more glycolytic type of muscle metabolism typically for fast twitch PM [67,68].

Interestingly, maximum values of RNA/DNA ratio as well as DNA and RNA concentration per gram of muscle were found at day five in control and EF-supplemented chicks whereas protein content and CK activity, have increased continuously over the experimental period. This could reflect that SATC/myoblast proliferative activity and the number of SATC per gram muscle is highest at hatching and reaches its peak between days 2 and 4 of life [37,39,41] whereas myogenic processes (differentiation, protein accretion) increase further on. This idea is also supported by lower DNA/protein and RNA/protein ratios at day 8 and 12 compared with day 1 and 5 of age. Interestingly, data from Halevy et al. [39,46] showed that GH-R-mediated inhibition of differentiation by chicken growth hormone is responsible for the high proliferation rate of SATC directly post-hatch.

Typically, SATC mitotic activity declines rapidly after hatching [41]. For example, mitotic activity at day seven after hatch was reduced to one-third compared to that seen at day one [37]. Indeed, in both groups, the number of total nuclei per mm^2^ decreased significantly from day five on.

Nevertheless, in our study, myonuclei number per myofiber and FCSA increased over the complete experimental period in both groups. However, compared with controls, an elevated number of myonuclei per fiber was observed in EF chicks from day five on. One explanation for these results comes from our data showing a higher *IGF1* mRNA expression in muscle samples from EF-supplemented compared with Con chicken. As shown for other species [47], IGF-I exerts positive effects on multiplication, growth and differentiation of SATC, myoblasts, and myotubes originating from chicken [69,70,71]. Our data indicate, that due to higher *IGF1* expression in PM of EF-treated chicks more SATC escape terminal differentiation and proliferate for a longer time, thereby increasing the SATC pool size and the number of myonuclei per fiber over those of control animals. In both groups, an age depending decrease of intramuscular *IGF1* mRNA expression was observed which step by step will increase the myogenic potential [47]. Induction of the myogenic regulatory factor *MYOG* is an essential component of IGF-I-dependent initiation of terminal differentiation [72,73]. *MYOG* expression is needed for the generation of fusion competent myoblasts that can supply new DNA to growing fibers [74], but its expression has not been investigated in our study. Nevertheless, at day 12 of life, the FCSA was larger in the EF group probably reflecting an increased fusion potential of *MYOG*-expressing cells [35].

Instead of the differentiation factor *MYOG* [45,74], we investigated the mRNA expression of the determination gene *MYF5*. *MYF5* primes SATC for myogenic commitment thereby preventing them from adopting other than the myogenic fate [45,74]. Although *MYF5* is expressed (mRNA and protein) in quiescent SATC/reserve cells and SATC-derived myoblasts it regulates myogenic process independently of *PAX7* [43,74,75]. In the present study, we found in both groups a low variability in the *MYF5* mRNA expression level over time. However, the expression level was generally higher in controls pointing to a higher proportion of cells primed for differentiation in the total SATC population of this group [42]. The lower level of *MYF5* expression in the EF group was unexpected and, at first sight, it does not fit with data showing that *PAX7* directly regulates the expression of *MYF5* mRNA and protein [76]. Gayraud-Morel et al. [43] investigated the commitment and self-renewal potential of SATC with one compromised *MYF5* allele (*MYF5* heterozygous cells) and a 50% reduction of MYF5 levels compared to the wild type SATC. Quiescent *MYF5* heterozygous cells express normal *PAX7* levels but had a more committed status as reflected by expression of *MYOD*, *MYOG* and the adult structural protein Troponin-T. Interestingly, transplantation in regenerating muscle reveals a higher self-renewing capacity in *MYF5* heterozygous compared with normal SATC. It seems thus possible that low *MYF5* expressing SATC from the EF group show similar properties, which is in accord with our observation of more PAX7+ cells. Angiopoietin and its receptor Tie2 have been shown to downregulate *MYF5* in SATC [77]. Both are expressed by SATC [43,77] but can also be provided by perivascular or interstitial cell types. Interestingly, compared with controls, *PAX7* mRNA levels are upregulated in muscle samples from EF-treated chicks at day 12 post-hatch. *PAX7* has essential functions in early postnatal/post-hatch development of SATC [41,42]. During this period, PAX7 is required for the expansion and survival of muscle progenitor cells, and for maintenance of their myogenic potential but it also regulates the transition from cycling muscle progenitor cells to the adult, quiescent SATC status [40,78]. From our mRNA expression data, we cannot exclude that higher *PAX7* levels only promote the latter process. Therefore, we also quantified the number of PAX7 positive cells present in *pectoralis* muscle cross sections of 12-day-old control and EF-treated chicks. The proportion of PAX7+ to total nuclei is in accord with data from Halevy et al. [41] and amounts to about 10%, with a trend for higher levels in the EF group. More importantly, EF-treated chicks had more myonuclei per fiber and more of them were PAX7+ compared with controls. Halevy et al. [41] have shown that the majority of PAX7+ cells are in the pre-myogenin state during this developmental stage. However, most cells co-express PAX7 and MYOD (Pax7+/MyoD+) and the ratio between them is critical for the fate determination of myoblasts [79]. MYOD is found in myoblasts and drives them into the next steps of differentiation. A higher expression of PAX7 has been shown to delay or prevent the expression of *MYOG* thereby favoring proliferation (and survival) of SATC over terminal differentiation [45,79], which is in agreement with our results for 12-day-old EF-treated chicks.

The early post-hatch period is not only critical for myofiber growth but also for tissue vascularization, which is rudimentary at hatching. To ensure normal muscle development, both processes must develop in parallel [80] and are regulated by common factors [81,82,83]. By secreting proangiogenic growth factors such as the master driver VEGF (vascular endothelial growth factor), HGF, FGF as well as HIF, SATC play an important role in the initiation of the angiogenic program. Therefore, it was not surprising that in our previous study [49], disturbed SATC function induced by early post-hatch SE infection led to significantly reduced myofiber growth and strong negative effects on capillarization. Interestingly, application of EF could not prevent negative effects of SE infection on FCSA growth, but completely restored normal capillarization [49]. Here, we observed positive effects of EF treatment on both, hypertrophic myofiber growth and capillarization that manifests at day 8 and/or day 12 of age. Capillary supply decreased with age in Con while an increase was found in EF chicks. As was found by others [84], capillary density decreases with age in both groups. However, the area covered by capillaries (day 8 and day 12) and the CCSA (day 12) were higher in EF chicks compared with controls. Myofiber diameters increased with age in both groups but at day 12 EF-treated chicks had lager fibers. Large myofiber diameters are speculated to lead to metabolic stress in fast growing broilers muscles such as PM as they are associated with longer diffusion distances for O_2_, nutrients and metabolites such as lactate. Larger capillaries as in EF treated chicks will provide an increased exchange surface. Possibly, this will help to reduce or to overcome negative consequences of fast myofiber growth such as hypoxia, increased oxidative stress and reduced intramuscular pH values. These factors are thought to increase the risk of myopathies particularly in animals with very high weight gain during the first two weeks after hatch [85,86].

## 5. Conclusions

The probiotic EF AL41 when applied to chicks for seven days after hatching seems to have positive effects on myofiber growth but also improves the capillarization needed for supplying adequate amounts of O_2_, nutrients, endocrine, and growth factors, as well as the removal of metabolic waste. Thus, it could be a good option to achieve reliable positive effects on broiler health and performance without increasing their susceptibility to myopathies.

## Figures and Tables

**Table 1:** List of primers used for the chicken mRNA quantification.

Primer	Sequence 5′–3′	Annealing Temperature/Time	References
*IGF1* Fw	GAGCTGGTTGATGCTCTTCAGTT	60 °C/1 min	Xiao et al., 2017 [58]
*IGF1* Rev	CCAGCCTCCTCAGGTCACAACT		
*MYF5* Fw	CAGAGACTCCCCAAAGTGGAGAT	60 °C/1 min	
*MYF5* Rev	GTCCCGGCAGGTGATAGTAGTTC		
*PAX7* Fw	AGGCTGACTTCTCCATCTCTCCT	60 °C/1 min	Adhikari et al., 2019 [59]
*PAX7* Rev	TGTAACTGGTGGTGCTGTAGGTG		
*GAPDH* Fw	CCTGCATCTGCCCATTT	59 °C/30 s	De Boever et al., 2008 [60]
*GAPDH* Rev	GGCACGCCATCACTATC		

**Table 2:** Daily weight gain, body weight, muscle weight and organ development of control and *E. faecium* AL41 (EF)-treated chicks between day 1 and 12 of life.

	Control				EF						*p*-Value		
Day of Age	1 (*n* = 6)	5 (*n* = 10)	8 (*n* = 10)	12 (*n* = 10)	1 (*n* = 6)	5 (*n* = 10)	8 (*n* = 10)	12 (*n* = 10)	SE d 1	SE d 5–12	Age	Group	Age × Group
Daily gain, g		14.0 ^c^	22.8 ^a^	17.3 ^b^		14.9 ^c^	24.1 ^a^	18.4 ^b^		0.39	<0.001	<0.001	0.919
Weights, g													
Slaughter weight	43.2 ^d^	112.7 ^c^	224.4 ^b^	250.5 ^a,B^	43.8 ^d^	116.8 ^c^	233.1 ^b^	261.4 ^a,A^	4.0	3.1	<0.001	0.012	0.456
*Pectoralis* muscle	1.74 ^d^	8.26 ^c^	35.66 ^b^	58.54 ^a,B^	1.64 ^d^	8.19 ^c^	37.21 ^b^	62.63 ^a,A^	1.39	1.07	<0.001	0.100	0.212
Liver	2.36 ^c^	7.47 ^b,A^	11.32 ^a^	12.46 ^a,A^	1.69 ^c^	4.50 ^b,B^	10.67 ^a^	11.21 ^a,B^	0.54	0.42	<0.001	<0.001	0.030
Stomach	5.01 ^b^	4.77 ^b,B^	12.34 ^a^	11.31 ^a^	4.32 ^b^	6.53 ^b,A^	11.14 ^a^	11.64 ^a^	0.60	0.46	<0.001	0.901	0.014
Intestine	5.62 ^c^	9.88 ^b^	21.61 ^a^	24.46 ^a^	4.90 ^c^	9.28 ^b^	22.13 ^a^	22.39 ^a^	1.04	0.80	<0.001	0.246	0.464

^a–d^ Different lower case letters indicate significant differences among ages (*p* < 0.05). ^A,B^ Different upper case letters indicate significant differences between groups (*p* < 0.05).

**Table 3:** Changes of DNA, RNA, and protein contents and concentrations as well as enzyme activities in *pectoralis* muscle of control and *E. faecium* AL41 (EF) supplemented chicks during day 1 to 12 of age.

	Control				EF						*p*-Value		
Day of Age	1 (*n* = 6)	5 (*n* = 10)	8 (*n* = 10)	12 (*n* = 10)	1 (*n* = 6)	5 (*n* = 10)	8 (*n* = 10)	12 (*n* = 10)	SE d 1	SE d 5–12	Age	Group	Age × Group
Total DNA, mg	0.48 ^d^	3.03 ^c^	7.72 ^b^	11.38 ^a^	0.37 ^d^	2.95 ^c^	7.58 ^b^	12.36 ^a^	0.34	0.27	<0.001	0.435	0.123
Total RNA, mg	2.54 ^d^	20.42 ^c^	48.56 ^b^	59.78 ^a,B^	2.16 ^d^	19.80 ^c^	44.34 ^b^	67.04 ^a,A^	1.96	1.52	<0.001	0.660	0.003
Total protein, mg	80 ^c^	501 ^c^	2551 ^b^	4620 ^a^	78 ^c^	482 ^c^	2753 ^b^	4961 ^a^	131	102	<0.001	0.098	0.276
DNA/RNA	0.188 ^a,A^	0.148 ^b^	0.159 ^b,B^	0.191 ^a^	0.170 ^b,B^	0.149 ^c^	0.171 ^b,A^	0.184 ^a^	0.003	0.003	<0.001	0.138	<0.001
RNA/DNA	5.32 ^c,B^	6.76 ^a^	6.28 ^b,A^	5.25 ^c^	5.93 ^b,A^	6.74 ^a^	5.83 ^bc,B^	5.44 ^c^	0.11	0.09	<0.001	0.249	<0.001
DNA, µg/g	273 ^b,A^	366 ^a^	217 ^c^	195 ^d^	226 ^b,B^	360 ^a^	204 ^c^	197 ^c^	5	4	<0.001	<0.001	<0.001
RNA, µg/g	1453 ^b^	2474 ^a^	1362 ^b,A^	1024 ^c^	1340 ^b^	2423 ^a^	1192 ^bc,B^	1071 ^c^	44	34	<0.001	0.008	0.020
Protein, mg/g	45.9 ^d^	60.7 ^c^	71.6 ^b^	79.0 ^a^	47.3 ^c^	58.8 ^c^	74.0 ^b^	79.1 ^a^	1.6	1.2	<0.001	0.588	0.327
DNA/protein, µg/mg	5.98 ^a,A^	6.04 ^a^	3.03 ^b^	2.46 ^c^	4.79 ^b,B^	6.13 ^a^	2.76 ^c^	2.50 ^c^	0.12	0.09	<0.001	<0.001	<0.001
RNA/protein, µg/mg	31.8 ^b^	40.8 ^a^	19.1 ^c,A^	12.9 ^d^	28.4 ^b^	41.3 ^a^	16.1 ^c,B^	13.6 ^c^	0.8	0.6	<0.001	0.009	0.004
Protein/RNA, mg/µg	0.032 ^c^	0.025 ^c^	0.053 ^b,B^	0.077 ^a^	0.036 ^c^	0.024 ^d^	0.063 ^b,A^	0.074 ^a^	0.002	0.001	<0.001	0.019	<0.001
Total CK, IU	1591 ^c^	13,347 ^c^	65,827 ^b^	116,724 ^a^	1612^c^	12,557 ^c^	67,609 ^b^	126,644 ^a^	3553	2752	<0.001	0.198	0.218
CK, IU/g	919 ^c^	1622 ^b^	1845 ^ab^	1994 ^a^	968 ^c^	1540 ^b^	1822 ^a^	2026 ^a^	70	54	<0.001	0.882	0.668
CK/protein, IU/mg	20.2 ^b^	26.8 ^a^	25.7^a^	25.2^a^	20.4^b^	26.3^a^	24.6 ^a^	25.7 ^a^	1.0	0.8	<0.001	0.703	0.751
ICDH, IU/g	3.12 ^a^	2.28 ^b^	1.41 ^c^	1.25 ^c^	2.96 ^a^	2.31 ^b^	1.39 ^c^	1.13 ^c^	0.09	0.07	<0.001	0.218	0.533
LDH, IU/g	43 ^d^	330 ^c^	751 ^b^	929 ^a^	54 ^d^	315 ^c^	765 ^b^	892 ^a^	29	22	<0.001	0.694	0.658
ICDH/protein, IU/mg	0.068 ^a^	0.038 ^b^	0.020 ^c^	0.016 ^c^	0.062 ^a^	0.039 ^b^	0.019 ^c^	0.014 ^c^	0.001	0.001	<0.001	0.070	0.040
LDH/protein, IU/mg	0.93 ^d^	5.42 ^c^	10.5 ^b^	11.74 ^a^	1.14 ^c^	5.36 ^b^	10.34 ^a^	11.28 ^a^	0.32	0.25	<0.001	0.536	0.695
LDH/ICDH	14 ^d^	146 ^c^	540 ^b^	748 ^a^	19 ^d^	138 ^c^	554 ^b^	796 ^a^	29	23	<0.001	0.394	0.660

^a–d^ Different lower case letters indicate significant differences among ages (*p* < 0.05). ^A,B^ Different upper case letters indicate significant differences between groups (*p* < 0.05).

**Table 4:** Muscle structure of *pectoralis* muscle in untreated control and *E. faecium* AL41 (EF) supplemented chicks between 5 and 12 d of age.

	Control				EF						*p*-Value		
Day of Age	1 (*n* = 6)	5 (*n* = 10)	8 (*n* = 10)	12 (*n* = 10)	1 (*n* = 6)	5 (*n* = 9)	8 (*n* = 10)	12 (*n* = 10)	SE d 1	SE d 5–12	Age	Group	Age × Group
Nuclei/mm^2^	7.622 ^c,A^	2.689 ^b,B^	1.648 ^a^	1.198 ^a^	6.515 ^c,B^	3.356 ^b,A^	1.803 ^a^	1.142 ^a^	257	199	<0.001	0.580	0.004
FCSA, µm^2^	54.5 ^d^	227.7 ^c^	577.5 ^b^	816.1 ^a,B^	67.9 ^d^	220.6 ^c^	586.4 ^b^	1107.8 ^a,A^	48.9	37.9	<0.001	0.011	<0.001
Nuclei/fiber	0.40 ^d^	0.57 ^c,B^	0.92 ^b,B^	0.95 ^a,B^	0.43 ^c^	0.71 ^b,A^	1.04 ^a,A^	1.23 ^a,A^	0.05	0.04	<0.001	<0.001	0.021
CCSA, µm^2^		3.59 ^c^	10.02 ^b^	13.31 ^a,B^		4.77 ^c^	11.78 ^b^	22.44 ^a,A^		0.88	<0.001	<0.001	<0.001
Capillary area, %		2.29	2.21 ^B^	1.92 ^B^		2.41	3.01 ^A^	2.89 ^A^		0.22	0.461	0.001	0.141
Capillaries/mm^2^		6496 ^a,A^	2162 ^b^	1476 ^b^		5246 ^a,B^	2616 ^b^	1324 ^c^		222	<0.001	0.091	0.002
Capillaries/fiber		1.50 ^A^	1.30	1.29		1.01 ^B^	1.39	1.45		0.13	0.656	0.453	0.033

^a–d^ Different lower case letters indicate significant differences among ages (*p* < 0.05). ^A,B^ Different upper case letters indicate significant differences between groups (*p* < 0.05). FCSA, fiber cross sectional area; CCSA, capillary cross sectional area.

## Data Availability

The raw data were generated at the FBN Dummerstorf (Germany), the Research Institute for Animal Production, Nitra and the University of Veterinary Medicine and Pharmacy, Košice (Slovakia). Derived data supporting the findings of this study are available from the corresponding author on request.
